# Fewer Minor Modified Duke Criteria on Admission Are Associated with Worse 90-Day Mortality in Patients with Confirmed Infective Endocarditis

**DOI:** 10.3390/jcm14217703

**Published:** 2025-10-30

**Authors:** Felix von Sanden, Kathrin Orlovius, Stefanie Andreß, Jonathan Ihrig, Friederike Schröder, Armin Imhof, Dominik Buckert, Wolfgang Rottbauer, Sascha d’Almeida

**Affiliations:** 1 Department of Internal Medicine II, Cardiology, Angiology, Pneumology, Internal Intensive Care, Sports and Rehabilitation Medicine, Ulm University Medical Center, 89081 Ulm, Germanysascha.almeida@uniklinik-ulm.de (S.d.); 2 Department for Anesthesiology and Intensive Care Medicine, Ulm University Medical Center, 89081 Ulm, Germany

**Keywords:** infective endocarditis, diagnostic challenges, mortality, minor duke criteria

## Abstract

**Background/Objectives**: Timely diagnosis of infective endocarditis (IE) remains a significant challenge, and IE poses significant morbidity and mortality. Modified Duke criteria (MDC) are used for the clinical evaluation and diagnosis of IE, but their current use is dichotomous. There are no studies that associate the amount of positive MDC with the patient’s outcome. This study intends to analyze whether the amount of MDC on initial presentation can be used for prognostic assumptions. **Methods**: We conducted a retrospective data analysis on patients with confirmed and suspected IE who were treated at the Department of Internal Medicine II at Ulm University Heart Center from December 2009 to December 2019. Univariable and multivariable logistic regression models were used to find correlations between 90-day mortality and the number of MDC. **Results**: 130 patients with confirmed IE were included in the analysis. Less minor MDC (OR 1.718; 95%-CI 1.096–3.268; *p* = 0.022) and a history of coronary artery disease (OR 4.711; 95%-CI 1.791–12.393; *p* = 0.002) were independently associated with higher 90-day mortality in patients with ultimately confirmed IE. Fewer minor MDC on presentation were associated with later diagnosis (b 2.341; 95%-CI 0.312–4.370; *p* = 0.024) and antibiotic therapy (b 2.953; 95%-CI 0.82–5.084; *p* = 0.007) for IE. **Conclusions**: Early diagnosis of IE is essential for favorable outcomes. Fewer minor MDC on initial presentation may lead to delayed diagnosis, antibiosis, and worse outcomes.

## 1. Introduction

Infective endocarditis (IE) remains a significant clinical challenge characterized by high morbidity and mortality despite advances in diagnostic techniques and therapeutic interventions. IE, an infection of the endocardial surface of the heart, often involves heart valves and is associated with substantial complications such as heart failure, systemic embolism, and persistent infection [1,2]. Early and accurate diagnosis is critical to improve patients’ outcomes [3] yet remains complex due to the heterogeneity of clinical presentations and microbiological findings [4].

The modified Duke criteria (MDC), established in 2000 and subsequently updated, have become the cornerstone for diagnosing IE, integrating clinical, microbiological, and echocardiographic findings to stratify cases into definite, possible, or rejected categories [5]. These criteria are widely used both in clinical practice and research to standardize diagnosis and guide management strategies [6]. However, while their longer-term diagnostic utility is well established, the prognostic value of the modified Duke criteria at the time of hospital admission remains unknown.

Several studies have examined predictors of mortality in IE, including patient comorbidities, causative pathogens, and complications [7,8,9,10]. Although MDC have demonstrated prognostic value (by distinguishing confirmed from possible IE, with poorer prognosis in definitive cases) [11,12], it is still uncertain whether the total number of MDC, particularly in the early phase of endocarditis, has independent prognostic value.

In this study, we aim to investigate whether classification according to the MDC at the time of hospital admission influences 90-day mortality in patients with confirmed IE. Understanding this association could provide valuable insights into early prognostication and tailor therapeutic approaches to reduce mortality in this high-risk population.

## 2. Materials and Methods

### 2.1. Study Design

We conducted a retrospective data analysis on patients with confirmed and suspected IE who were treated at our Department of Internal Medicine II at Ulm University Heart Center from December 2009 to December 2019. In compliance with established European Society of Cardiology (ESC) guidelines, the diagnosis of IE followed the assessment of MDC. MDC were assessed on initial admission, either at our university medical center or at the transferring hospital and were subsequently reassessed during the hospital stay at the discretion of the attending physician. Blood cultures, which were drawn within 24 h of admission and provided evidence for microbial growth in the following days, were counted towards MDC on admission. Echocardiographic findings were counted towards MDC on admission, if obtained within 24 h of admission. IE was confirmed with either two positive major MDC, one positive major MDC and at least three positive minor MDC, or five positive minor MDC during the clinical course. Patients with possible or rejected IE were not included in this study. The primary outcome was a 90-day mortality after diagnosis of IE.

### 2.2. Statistical Analysis

Data analyses were performed using SPSS (version 28.0, IMB Corporation, Armonk, NY, USA). Categorical variables were expressed as absolute (*n*/*N*) and relative frequencies (%). Continuous variables were expressed as means ± standard deviation (SD) or medians and interquartile range (IQR), depending on the distribution. The normality of distribution was assessed by visual analysis of plotted histograms as well as Shapiro–Wilk and Kolmogorov–Smirnov tests. Pearson’s chi-squared tests were used to unveil statistically significant differences in values between categorial variables. If not otherwise defined, all comparisons refer to the average of the included subgroups.

Univariable and backwards stepwise multivariable logistic regression models were used to identify parameters associated with 90-day mortality in patients with confirmed IE. Variables with *p*-values less than 0.10 in univariable analyses were considered in the multivariable analysis (entry-threshold, *p* = 0.05; removal-threshold *p* = 0.10). To assess multicollinearity among predictor variables, we calculated variance inflation factor (VIF) for each variable before inclusion to the multivariable analysis. This involves fitting a linear regression model where one predictor is regressed on all the other predictors. A VIF of three or less was considered acceptable for inclusion to the multivariable analysis.

Univariable linear regression models were used to find significant mediating factors contributing to the observed outcome. Box-Plots were used for illustration.

*p*-values < 0.05 were considered significant for all tests.

## 3. Results

### 3.1. Patient Characteristics

Patient characteristics are shown in Table 1. In total, 130 patients (96 male, 73.8%) were included in the study. The median age on admission was 66.0 (55.3–75.8) years, the mean body height was 173.3 ± 9.0 cm, the median body mass was 80.0 (67.8–90.0) kg, and the median body mass index was 25.9 (23.2–29.4) kg/m^2^. Of the 130, 62 Patients (47.7%) were transferred from another hospital.

### 3.2. Modified Duke Criteria on Admission and at Time of Diagnosis

Table 2 summarizes the number of MDC assessed on admission. Patients who were admitted at a different hospital and transferred to our institution presented less frequently with no major MDC (17.7% vs. 55.9%, *p* < 0.001) and more often with two major MDC (43.5% vs. 8.8%, *p* < 0.001). There were no significant differences concerning the number of minor MDC on admission in regards of the admission site. Overall, the diagnosis of IE would have been rejected in 51 patients (39.2%) if only the information acquired on the day of admission was considered.

The individual numbers of major and minor MDCs on admission and the time of diagnosis are shown in Table 3. Less than half of patients presented with echocardiographic evidence of IE or had positive blood cultures for IE, which were drawn on admission. The most frequent minor criteria on admission were predisposing factors, followed by temperature over 38 °C, and vascular phenomena. Over the clinical course, an increase in both major criteria could be observed (*p* < 0.001). Furthermore, vascular and immunologic phenomena were observed significantly more often at the time of diagnosis of IE.

### 3.3. Outcome and Site of Infective Endocarditis

The most common site for endocardial infection was the aortic valve, followed by the mitral valve. Infections of the tricuspid valve were less frequent and only one patient had been diagnosed with IE of the pulmonary valve. Of the included 28 patients with an implanted pacemaker or ICD, 15 had an infection of the implanted device and/or a lead infection. Twenty-six patients (20.0%) died within 90 days of initial diagnosis of IE. Of the patients, 52.3% received treatment in the intensive care unit. Surgery for IE was undertaken in 41.5% of patients. In 33 of the 37 patients with prosthetic heart valves, IE involved at least one valve prosthesis. Table 4 summarizes patients’ outcomes and sites of IE.

### 3.4. Clinical Parameters Associated with 90-Day Mortality

Univariable and multivariable logistic regression analyses are summarized in Table 5. As all VIFs were lower than 3, there was no significant evidence for multicollinearity and no variables had to be excluded from multivariable analysis. Clinical variables associated with the 90-day-mortality for patients with ultimately confirmed IE in multivariable analysis were a history of coronary artery disease (OR, 4.711; 95% CI, 1.791–12.393; *p* = 0.002) and fewer minor MDC on admission (OR, 1.718; 95% CI, 1.096–3.268; *p* = 0.022). Age, diabetes mellitus, and a history of myocardial infarction did correlate with the 90-day mortality in univariable analysis but had no independent multivariable analysis. Major MDC on admission, body height, body weight, gender, left ventricular function, site of infection, or history of cancer did not affect the 90-day mortality in our logistic regression model.

### 3.5. Distribution of Modified Duke Criteria on Admission and 90-Day Mortality

Table 6 shows a cross table illustrating the distribution of major and minor MDC on admission among our patient collective with ultimately confirmed IE, as well as the individual absolute and relative frequencies of 90-day mortality. The highest relative incidence was observed in the group with neither major nor minor MDC upon initial assessment on admission. No patients died within 90 days who presented with two major and three minor MDC on admission. Also, the singular patient with four minor and one major MDC survived for at least 90 days.

### 3.6. Factors Associated with Fewer Minor Modified Duke Criteria on Admission

**Figure 1:**
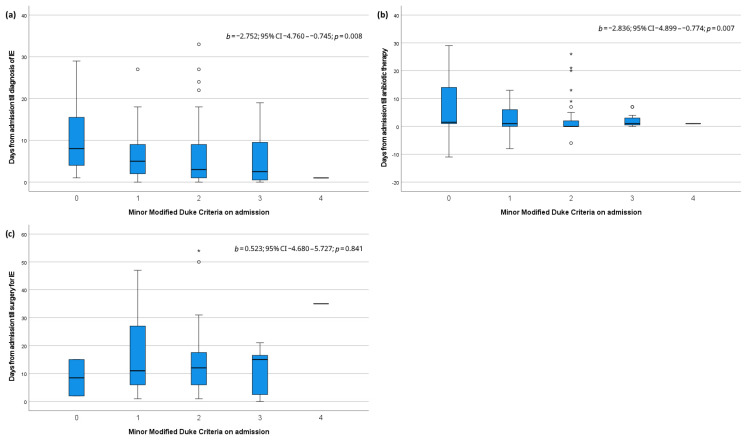
Box plots and linear regression analysis for minor modified Duke criteria on admission and (**a**) days till diagnosis of infective endocarditis, (**b**) days till antibiotic therapy and (**c**) days till surgery for infective endocarditis. IE: infective endocarditis, °: mild outliers, *: extreme outliers.

Box plots illustrating the relationship of minor MDC on admission with the time till diagnosis, antibiotic therapy, and surgery for IE are shown in Figure 1. Linear regression showed that the assessed number of minor MDC on admission correlated inversely and significantly with the time in days from admission to diagnosis of IE (b −2.752; 95% CI −4.760–−0.745; *p* = 0.008) and initialization of antibiotic therapy (b −2.836; 95% CI −4.899–−0.774; *p* = 0.007). The timing of surgery for IE was not affected by the number of minor MDC.

## 4. Discussion

Our results demonstrate that both fewer minor MDC on admission and pre-existing coronary artery disease are significant independent negative prognostic factors, which contribute to an increased 90-day mortality in patients with IE.

Although the highest relative 90-day mortality was observed in patients without major or minor MDC on admission, our findings suggest that patients with fewer minor MDC on admission tend to have worse 90-day mortality, independent of the presence of major MDCs on admission.

Furthermore, our study showed a correlation between fewer minor MDC on admission with a delay in diagnosis and antibiotic therapy. Patients with low MDC may present with subclinical or ambiguous symptoms, potentially leading to a less thorough investigation for IE and complication of the clinical decision-making process. Previous studies have described a high frequency of initially negative transthoracic echocardiogram examinations in 42% of cases with ultimately confirmed endocarditis [13]. If the suspicion of IE is waived due to initially negative MDC, a delay in diagnosis, sufficient antibiotic therapy, and surgery (if indicated) can lead to worse outcomes [3]. In our study cohort of 130 patients with ultimately confirmed IE, only 42.3% had echocardiographic evidence for IE on admission, and in 45.4% of cases, blood cultures drawn on admission later turned positive. Additionally, only 13.1% of patients had three or more minor MDC and no patient had five on admission. Considering that our study exclusively included patients with ultimately confirmed IE, relying solely on data available at admission would thus have theoretically resulted in a misclassification as ‘rejected’ in 39.2% of cases, highlighting the limited early sensitivity of the modified Duke criteria [14].

Patients who had been transferred from another hospital for specialized treatment more frequently presented with two major MDCs on initial presentation compared to patients admitted directly at our center. This may be attributed to selection or referral bias, as these patients had an early diagnosis of IE, which may have prompted preferred transfer to our center. Early positive blood cultures for infective endocarditis, particularly those with a short time to positivity, may indicate more severe disease, which is linked to worse outcomes and higher mortality [15].

Although there are currently no studies directly linking a history of coronary artery disease with worse outcomes in IE, acute coronary artery syndromes are linked with worse clinical outcome and mortality [16,17,18]. Furthermore, previous cardiac surgery, a potential surrogate for an underlying coronary artery disease, is linked with worse outcomes in patients with IE [19].

In cases of acute myocardial infarction and percutaneous coronary artery intervention, the inflammatory markers are often elevated, and fever may also develop as part of the systemic inflammatory response [20,21,22]. These clinical signs can mimic those seen in IE, potentially leading to a delay in diagnosis, despite the presence of clinical features that would otherwise prompt further investigation for IE.

Our study indicated that even in cases with a low suspicion for cardiac infections or in the presence of alternative explanations for symptoms, it is essential to actively investigate for IE as an insufficient early search for MDC may lead to delayed or false-negative findings [23].

### Study Limitations

This study was retrospective in nature, and, thus, general limitations for this study design apply. A major limitation of this study is the small sample size, which may have led to type II errors. A selection bias favorable to a patient cohort with more complex disease could occur, as the study was carried out in a highly specialized tertiary care center.

## 5. Conclusions

With the limitations of a retrospective study design and a small patient cohort, fewer minor MDC on admission and a history of coronary artery disease presented as independent risk factors for an increased 90-day mortality in patients with ultimately confirmed IE. Fewer minor MDC on presentation were potentially associated with delayed diagnosis and antibiotic therapy, indicating diagnostic difficulties in this complex patient cohort.

## Figures and Tables

**Table 1:** Baseline characteristics on the time of admission.

Age [median (IQR)]	66.0 (55.3–75.8)
Male gender [*n*/*N* (%)]	96/129 (73.8%)
Body height in cm [mean ± SD]	173.3 ± 9.0
Body mass in kg [median (IQR)]	80.0 (67.8–90.0)
BMI in kg/m^2^ [median (IQR)]	25.9 (23.2–29.4)
Arterial hypertension [*n*/*N* (%)]	83/130 (63.8%)
Type I diabetes [*n*/*N* (%)]	2/130 (1.5%)
Type II diabetes [*n*/*N* (%)]	37/130 (28.5%)
Peripheral artery disease [*n*/*N* (%)]	17/130 (13.1%)
Coronary artery disease [*n*/*N* (%)]	55/130 (42.3%)
Myocardial infarction [*n*/*N* (%)]	21/130 (16.2%)
Impaired LV-function [*n*/*N* (%)]	57/113 (50.4%)
Structural heart defect [*n*/*N* (%)]	9/130 (6.9%)
History of cancer [*n*/*N* (%)]	12/130 (9.2%)
Dialysis at baseline [*n*/*N* (%)]	6/130 (4.6%)
Transfer from another hospital [*n*/*N* (%)]	62/130 (47.7%)
Valve prosthesis [*n*/*N* (%)]	37/130 (28.5%)
Pacemaker/ICD [*n*/*N* (%)]	28/130 (21.5%)

BMI: body mass index, ICD: implantable cardioverter-defibrillator, IQR: interquartile range, LV: left ventricular, *n*/*N* (%): absolute and relative frequency, SD: standard deviation.

**Table 2:** Number of modified Duke criteria on admission (total and separated by site of admission).

	Total	Internal Admission	Transferal	*p*-Value
0 Major MDC [*n*/*N* (%)]	49/130 (37.7%)	38/68 (55.9%)	11/62 (17.7%)	<0.001 [χ^2^]
1 Major MDC [*n*/*N* (%)]	48/130 (36.9%)	24/68 (35.3%)	24/62 (38.7%)	0.687 [χ^2^]
2 Major MDC [*n*/*N* (%)]	33/130 (25.4%)	6/68 (8.8%)	27/62 (43.5%)	<0.001 [χ^2^]
0 Minor MDC [*n*/*N* (%)]	13/130 (14.6%)	7/68 (10.3%)	12/62 (19.4%)	0.144 [χ^2^]
1 Minor MDC [*n*/*N* (%)]	42/130 (32.3%)	27/68 (39.7%)	15/62 (24.2%)	0.059 [χ^2^]
2 Minor MDC [*n*/*N* (%)]	52/130 (40.0%)	26/68 (38.2%)	26/62 (41.9%)	0.667 [χ^2^]
3 Minor MDC [*n*/*N* (%)]	16/130 (12.3%)	8/68 (11.8%)	8/62 (12.9%)	0.844 [χ^2^]
4 Minor MDC [*n*/*N* (%)]	1/130 (0.8%)	0/68 (0.0%)	1/62 (1.6%)	0.293 [χ^2^]

MDC: modified Duke criteria, *n*/*N* (%): absolute and relative frequency, χ^2^: Pearson’s chi-squared.

**Table 3:** Modified Duke criteria on admission and at time of diagnosis.

	Admission	Time of Diagnosis	*p*-Value
Major MDC			
Blood cultures positive for IE [*n*/*N* (%)]	59/130 (45.4%)	91/130 (70.0%)	<0.001 [χ^2^]
Echocardiographic evidence for IE [*n*/*N* (%)]	55/130 (42.3%)	120/130 (92.3%)	<0.001 [χ^2^]
Minor MDC			
Temperature > 38 °C [*n*/*N* (%)]	67/130 (51.5%)	79/130 (60.8%)	0.133 [χ^2^]
Predisposing factors [*n*/*N* (%)]	78/130 (60.0%)	78/130 (60.0%)	1.000 [χ^2^]
Vascular phenomena [*n*/*N* (%)]	38/130 (29.2%)	69/130 (53.1%)	<0.001 [χ^2^]
Immunologic phenomena [*n*/*N* (%)]	4/130 (3.1%)	15/130 (11.5%)	0.009 [χ^2^]
Microbiologic evidence [*n*/*N* (%)]	11/130 (8.5%)	7/130 (13.1%)	0.230 [χ^2^]

IE: infective endocarditis, MDC: modified Duke criteria, *n*/*N* (%): absolute and relative frequency, χ^2^: Pearson’s chi-squared.

**Table 4:** Outcome and type of infective endocarditis.

90-day mortality after diagnosis [*n*/*N* (%)]	26/130 (20.0%)
Intensive care unit treatment [*n*/*N* (%)]	68/130 (52.3%)
Surgery for IE [*n*/*N* (%)]	54/130 (41.5%)
Aortic valve IE/*N* (%)]	59/130 (45.4%)
Pulmonary valve IE [*n*/*N* (%)]	1/130 (0.7%)
Mitral valve IE [*n*/*N* (%)]	56/130 (43.1%)
Tricuspid valve IE [*n*/*N* (%)]	11/130 (8.5%)
Native valve IE [*n*/*N* (%)]	84/130 (64.6%)
Prosthetic valve IE [*n*/*N* (%)]	33/37 (89.2%)
Device/Lead IE [*n*/*N* (%)]	15/28 (53.6%)

IE: infective endocarditis, *n*/*N* (%): absolute and relative frequency.

**Table 5:** Clinical parameters associated with 90-day mortality after diagnosis of endocarditis in univariable and multivariable logistic regression analysis.

	Univariable Analysis			Multivariable Analysis		
	OR	95%-CI	*p*-Value	OR	95%-CI	*p*-Value
Major MDC on admission [per one decrease]	1.355	0.771–2.387	0.291			
Minor MDC on admission [per one decrease]	1.669	1.018–2.732	0.042	1.718	1.096–3.268	0.022
Gender [male]	1.228	0.447–3.372	0.690			
Age [per 1-year increase]	1.039	1.006–1.073	0.022			
Body height [per 1 cm increase]	1.010	0.956–1.067	0.731			
Body weight [per 1 kg increase]	1.006	0.983–1.029	0.631			
BMI [per 1 kg/m^2^ increase]	1.042	0.970–1.119	0.259			
Referral from other hospital	1.361	0.575–3.223	0.483			
Dialysis on admission	0.792	0.089–7.087	0.835			
Arterial hypertension	2.169	0.803–5.858	0.127			
Diabetes type I or II	3.000	1.235–7.289	0.015			
Peripheral artery disease	1.273	0.378–4.283	0.697			
Coronary artery disease	4.074	1.616–10.270	0.003	4.711	1.791–12.393	0.002
History of myocardial infarction	3.111	1.127–8.589	0.028			
Reduced LV-function	1.100	0.426–2.841	0.844			
History of cancer	3.299	0.954–11.410	0.059			
Aortic valve IE	1.261	0.533–2.982	0.598			
Pulmonary valve IE	<0.001		1.0			
Mitral valve IE	0.788	0.327–1.899	0.586			
Tricuspid valve IE	0.376	0.046–3.078	0.376			
Device/Lead IE	0.833	0.137–5.007	0.843			
Prosthetic valve IE	0.545	0.340–3.619	0.482			

BMI: body mass index, IE: infective endocarditis, LV: left ventricular, MDC: modified Duke criteria, OR: odds ratio.

**Table 6:** Distribution of 90-day mortality in association with modified Duke criteria on admission.

90-Day Mortality *n*/*N* (%)	0 Minor MDC *N* = 19	1 Minor MDC *N* = 42	2 Minor MDC *N* = 52	3 Minor MDC *N* = 16	4 Minor MDC *N* = 1	Total *N* = 130
**0 Major MDC** ***N* = 49**	4/9 (44.4%)	4/18 (22.2%)	2/18 (11.1%)	1/4 (25.0%)	n/a	11/49 (22.4%)
**1 Major MDC** ***N* = 48**	2/6 (33.3%)	4/16 (25.0%)	4/20 (20.0%)	1/5 (20.0%)	0/1 (0.0%)	11/48 (22.9%)
**2 Major MDC** ***N* = 33**	1/4 (25.0%)	1/8 (12.5%)	2/14 (14.3%)	0/7 (0.0%)	n/a	4/33 (12.1%)
**Total**	7/19 (36.8%)	9/42 (21.4%)	8/52 (15.4%)	2/16 (12.5%)	0/1 (0.0%)	26/130 (20.0%)

MDC: modified Duke criteria, *n*: number of deceased patients after 90 days, *N*: number of Patients in group.

## Data Availability

The data presented in this study are available on request from the corresponding author. The data are not publicly available due to preservation of patient anonymity and privacy.

## Abbreviations

The following abbreviations are used in this manuscript:

BMI	Body mass index
ESC	European Society of Cardiology
IE	Infective endocarditis
IQR	Interquartile range
LV	Left ventricular
MDC	Modified Duke criteria
OR	Odds ratio
VIF	Variance inflation factor
